# Light-field tomographic fluorescence lifetime imaging microscopy

**DOI:** 10.21203/rs.3.rs-2883279/v1

**Published:** 2023-05-10

**Authors:** Yayao Ma, Luzhe Huang, Chandani Sen, Samuel Burri, Claudio Bruschini, Xilin Yang, Robert B. Cameron, Gregory A. Fishbein, Brigitte N. Gomperts, Aydogan Ozcan, Edoardo Charbon, Liang Gao

**Affiliations:** 1. Department of Bioengineering, University of California, Los Angeles, CA, USA; 2. Electrical and Computer Engineering Department, University of California, Los Angeles, CA, USA; 3. California Nano Systems Institute, University of California, Los Angeles, CA, USA; 4. UCLA Children’s Discovery and Innovation Institute, Mattel Children’s Hospital UCLA, Department of Pediatrics, David Geffen School of Medicine, UCLA, Los Angeles, CA, USA; 5. Advanced Quantum Architecture Laboratory, Ecole Polytechnique Federale de Lausanne, Neuchatel, Switzerland; 6. Department of Thoracic Surgery, University of California, Los Angeles, CA, USA; 7. Department of Pathology and Laboratory Medicine, David Geffen School of Medicine at UCLA, Los Angeles, CA, USA; 8. David Geffen School of Medicine, University of California Los Angeles, Los Angeles, CA, USA

## Abstract

Fluorescence lifetime imaging microscopy (FLIM) is a powerful imaging technique that enables the visualization of biological samples at the molecular level by measuring the fluorescence decay rate of fluorescent probes. This provides critical information about molecular interactions, environmental changes, and localization within biological systems. However, creating high-resolution lifetime maps using conventional FLIM systems can be challenging, as it often requires extensive scanning that can significantly lengthen acquisition times. This issue is further compounded in three-dimensional (3D) imaging because it demands additional scanning along the depth axis. To tackle this challenge, we developed a novel computational imaging technique called light field tomographic FLIM (LIFT-FLIM). Our approach allows for the acquisition of volumetric fluorescence lifetime images in a highly data-efficient manner, significantly reducing the number of scanning steps required compared to conventional point-scanning or line-scanning FLIM imagers. Moreover, LIFT-FLIM enables the measurement of high-dimensional data using low-dimensional detectors, which are typically low-cost and feature a higher temporal bandwidth. We demonstrated LIFT-FLIM using a linear single-photon avalanche diode array on various biological systems, showcasing unparalleled single-photon detection sensitivity. Additionally, we expanded the functionality of our method to spectral FLIM and demonstrated its application in high-content multiplexed imaging of lung organoids. LIFT-FLIM has the potential to open up new avenues in both basic and translational biomedical research.

Fluorescence lifetime imaging microscopy (FLIM) [1,2] has been extensively employed in a wide spectrum of biomedical applications, ranging from single cell studies [3–5] to medical diagnosis [6–8]. Rather than imaging the time-integrated fluorescent signals, FLIM measures the time-lapse fluorescent decay. Because the lifetime of a fluorophore is dependent on its molecular environment but not on its concentration, FLIM enables a more quantitative study of molecular effects inside living organisms compared with conventional intensity-based approaches.

The FLIM techniques are generally stratified into two categories: frequency-domain FLIM and time-domain FLIM. Frequency-domain FLIM utilizes frequency-modulated light to illuminate the sample, inducing fluorescence that oscillates at the same frequency but with a reduced modulation depth and a phase shift due to the non-instantaneous fluorescence decay [9]. To extract the phase shift and modulation depth from the fluorescence signals, frequency-domain FLIM modulates the gain of the camera at the same or slightly different frequency as the excitation light [10–13]. The fluorescence lifetime can then be derived from the measured signals by comparing them to a reference fluorophore with a known lifetime. In contrast, time-domain FLIM illuminates the sample with pulsed laser excitation, followed by measuring the fluorescent decay in sequential time channels using an ultrafast detector or detector array. Due to the direct measurement of the fluorescence decay, time-domain FLIM provides more accurate lifetime measurements than its frequency-domain counterpart, particularly for complex fluorophores that exhibit multiple decay components [14].

To acquire a two-dimensional (2D) lifetime map, most time-domain FLIM systems scan the sample either in the spatial domain, such as a confocal FLIM microscope [15,16], or in the time domain, such as a time-gated FLIM camera [17,18]. The spatial and lifetime resolution of the resulting map is determined by the number of scanning steps used in the respective domain. To acquire high-resolution images, conventional time-domain FLIM systems require extensive scanning, which can result in prolonged acquisition times. And this problem becomes more pronounced in three-dimensional (3D) imaging, as it mandates additional scanning along the depth axis.

The use of single-photon avalanche diode (SPAD) arrays in time-domain FLIM provides a solution to this long-standing problem by enabling parallel measurement of fluorescence decays at multiple image pixels. Moreover, SPAD arrays offer considerably greater sensitivity than conventional gated cameras, making them an excellent choice for low-light imaging applications. A SPAD imager can operate in either time gate or time-correlated single photon counting (TCSPC) mode, with TCSPC being the preferred detection method for its higher precision, faster speed, and greater sensitivity. However, the native fill factor of 2D SPAD arrays operating in the TCSPC mode is generally low (<10%) [19] due to the physical limitations posed by the inclusion of intricate timing electronics for each pixel. Although the addition of microlenses can recover some of the fill factor loss, this method is effective only for collimated impinging light. In contrast, a linear SPAD array offers a significantly higher fill factor close to 50% [20], which results in a substantially increased light throughput. Moreover, the fabrication cost of a linear SPAD array is much lower than its 2D counterpart, making it more accessible for general labs. Nonetheless, when it comes to high-resolution imaging of a 2D or 3D scene using a linear SPAD array system, the challenge is much like that of point-scanning FLIM—the conventional approach involves scanning the entire field of view or volume using a vast number of steps, which can result in a protracted imaging duration.

To tackle the aforementioned challenges and streamline the acquisition of 3D FLIM data using a linear SPAD array, we devised a novel computational imaging technique called LIght-Field Tomographic Fluorescence Lifetime Imaging Microscopy (LIFT-FLIM). Our approach has been only recently made possible by an emerging technique, light field tomography (LIFT) [21,22], which is highly efficient in acquiring light field data for 3D imaging. Sharing its roots with light field photography [23–25], LIFT acquires multiple views of a 3D object and determines depth information through disparity analysis. However, rather than directly capturing a 2D perspective image, LIFT measures only the en-face projections of the image, thereby transforming 2D perspective images into lines. This allows us to map high-dimensional optical information to a low-dimensional space through pure optical operations. In LIFT-FLIM, we take advantage of this transformation by directly capturing 1D projection images using a linear SPAD array, allowing for 3D fluorescence lifetime imaging with exceptional single-photon sensitivity.

Aside from its impressive 3D imaging capability, LIFT also possesses inherent compatibility with spectral imaging [26]. Capitalizing on this feature, we showcased the system’s versatility by extending its functionality to include spectral FLIM (sFLIM). We achieved this by dispersing the 1D projection images utilizing a diffraction grating, and then feeding the resulting image into a time-gated camera for precise lifetime measurement. This allows for the simultaneous acquisition of 3D FLIM images at multiple wavelengths, making our system a versatile tool for analyzing both lifetime and spectral information. We demonstrated LIFT-FLIM and LIFT-sFLIM on various biological systems and showed their potential for high-content multiplexed imaging.

## Results

### Operating principle and characterization

**Fig. 1.**
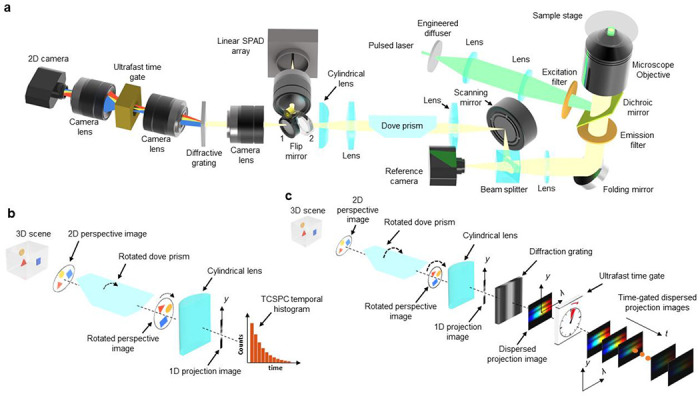
Optical setup and image formation models. **a.** System schematics. **b.** Image formation model of LIFT-FLIM. **c.** Image formation model of LIFT-sFLIM. SPAD, single-photon avalanche diode; TCSPC, time-correlated single photon counting.

Based on computational imaging, LIFT-FLIM operates in two steps: data acquisition and image reconstruction. We show a LIFT-FLIM system in Fig. 1a. Upon pulsed laser excitation, the fluorescence is collected by a microscope objective lens with a high numerical aperture (NA) and forms an intermediate image at the microscope’s side image port. A beam splitter then divides the fluorescence into two beams. The transmitted light is recorded directly by a complementary metal-oxide-semiconductor (CMOS) camera, resulting in a reference intensity image. On the other hand, the reflected light creates an intermediate image on a scanning mirror. As the mirror tilts, it imparts twice its angle of tilt onto the outgoing rays. The reflected light from the scanning mirror is then collimated by a lens and forms a pupil image at a plane, where we position a dove prism. By adjusting the tilt angle of the mirror, we can shift the position of the pupil image on this plane. This enables us to selectively direct the light rays corresponding to a specific view angle through the dove prism, which will rotate the light rays and produce a rotated perspective image (Fig. S1). Next, we use a cylindrical lens to compress the rotated perspective image into a line, which is essentially an en-face projection of the original perspective image along an orientation twice the rotation angle of the dove prism. This transformed line image can be either directly measured by a linear SPAD camera (Fig. 1b) or further spectrally dispersed and measured by a time-gated camera (Fig. 1c).

To compute a 3D image, conventional light field cameras require the acquisition of a comprehensive set of spatial and angular information of a light field, resulting in a significant data load. However, our previous work demonstrated that this acquisition method is inefficient and generates a substantial amount of redundant data [22], which can be reduced by distributing a nonlocal image acquisition process, such as en-face projection measurement, into different views. Moreover, this allows for the measurement of a high-dimensional light field using low-dimensional detectors, which are typically low-cost and feature a higher temporal bandwidth. In LIFT-FLIM, we leverage this advantage and capture only en-face 1D projection images at each scanned sub-pupil location. Furthermore, we can create an arbitrarily shaped off-focus point spread function (PSF) by strategically shuffling the orientation angles of our projection measurements across various views in a programmed manner (Supplementary Note 2).

We formulate the image formation of LIFT-FLIM using a linear model. For a given perspective image $P_{k}$ at view k(k=1,2,…,K), the projection measurement along angle θ is

(1) $${\text{f}}_{k}^{\theta }=TR^{\theta }{\text{P}}_{k},$$

where $P_{k}$ has a dimension of $N^{2}(N$ is the image dimension in pixels), T is the en-face projection operator, and $R^{\theta }$ is the image rotation operator, which describes the function of the dove prism rotated at θ/2.

Rather than capturing a complete set of $N_{\theta }=N$ projection angles at each view, we acquire only a subset of $n_{k}$ projection angles at view k. This process can be explicitly written as:

(2) $$\begin{array}{l} f=\left[\begin{array}{c} view\_1\{\left[\begin{array}{c} {\text{f}}_{\text{1}}^{{\text{θ}}_{\text{1}}}\\ {\text{f}}_{\text{1}}^{{\text{θ}}_{2}}\\ \vdots \\ {\text{f}}_{\text{1}}^{{\text{θ}}_{n_{1}}} \end{array}\right]\\ \vdots \\ view\_k\{\left[\begin{array}{c} {\text{f}}_{k}^{{\text{θ}}_{s_{k-1}+1}}\\ {\text{f}}_{k}^{{\text{θ}}_{s_{k-1}+2}}\\ \vdots \\ {\text{f}}_{k}^{{\text{θ}}_{s_{k-1}+n_{k}}} \end{array}\right]\\ \vdots \\ view\_K\{\left[\begin{array}{c} {\text{f}}_{K}^{{\text{θ}}_{s_{K-1}+1}}\\ {\text{f}}_{K}^{{\text{θ}}_{s_{K-1}+2}}\\ \vdots \\ {\text{f}}_{K}^{{\text{θ}}_{s_{K-1}+n_{K}}} \end{array}\right] \end{array}\right]\\ =T\left[\begin{array}{ccc} view\_1\{\left[\begin{array}{c} R^{\theta_{1}}\\ R^{\theta_{2}}\\ \vdots \\ R^{\theta_{n_{1}}} \end{array}\right] & \cdots & 0\\ \vdots & view\_k\{\left[\begin{array}{c} R^{\theta_{s_{k-1}+1}}\\ R^{\theta_{s_{k-1}+2}}\\ \vdots \\ R^{\theta_{s_{k-1}+n_{k}}} \end{array}\right] & \vdots \\ 0 & \ddots & 0\\ 0 & \ldots & view\_K\{\left[\begin{array}{c} R^{\theta_{s_{K-1}+1}}\\ R^{\theta_{s_{K-1}+2}}\\ \vdots \\ R^{\theta_{s_{K-1}+n_{K}}} \end{array}\right] \end{array}\right]\left[\begin{array}{c} {\text{P}}_{\text{1}}\\ {\text{P}}_{\text{1}}\\ \vdots \\ {\text{P}}_{K} \end{array}\right]+\sigma \\ =\left[\begin{array}{ccc} A_{1} & \cdots & 0\\ \vdots & A_{2} & \vdots \\ 0 & \ddots & 0\\ 0 & \cdots & A_{K} \end{array}\right]\left[\begin{array}{c} {\text{P}}_{\text{1}}\\ {\text{P}}_{\text{1}}\\ \vdots \\ {\text{P}}_{K} \end{array}\right]+\sigma . \end{array}$$

Here f is a stack of projection measurements (also referred to as a sinogram), and it has a dimension of $N\times N_{\theta }\;\cdot \;s_{k-1}=\sum_{i=1}^{i=k-1}\;n_{i}$, and $s_{K-1}+n_{K}=N_{\theta }\;\cdot \;\sigma$ denotes the measurement noise. $A_{k}$ is a combined function of en-face projection and image rotation operators on perspective image $P_{k}$. Because the images from different views capture the same scene, they share a common underlying content (Supplementary Note 1), with only a depth-dependent disparity between any two sub-aperture images, as shown in Fig. S1. Therefore, the correlation between sub-aperture images can be modeled by digitally propagating the light field, *i.e.* the sub-aperture image $P_{k}$ at view k can be related to a depth-dependent image feature kernel h(d) through an invertible shearing operator $B_{k}$ as $P_{k}=B_{k}(d)h(d)$, where $B_{k}$ is also a function of depth d (Supplementary Note 1). Accordingly, we transform Eq. 2 to:

(3) $$\text{f}=\left[\begin{array}{ccc} A_{1} & \cdots & 0\\ \vdots & A_{2} & \vdots \\ 0 & \ddots & 0\\ 0 & \cdots & A_{K} \end{array}\right]\;\left[\begin{array}{c} B_{1}(d)h(d)\\ B_{2}(d)h(d)\\ \vdots \\ B_{K}(d)h(d) \end{array}\right]+\sigma =\left[\begin{array}{c} A_{1}B_{1}(d)\\ A_{2}B_{2}(d)\\ \vdots \\ A_{K}B_{K}(d) \end{array}\right]h(d)+\sigma =F(d)h(d)+\sigma$$

The overall image forward operator F(d) becomes a function of depth d, which is essential for recovering image h(d) with various focal settings. Noteworthily, although individual $P_{k}$ is measured at only a subset of projection angles, the underlying image feature kernel h(d) is measured on a complete angular basis, as F(d) concatenates image rotation operators across all views.

For direct fluorescence lifetime measurement using a linear SPAD array with TCSPC (Fig. 1b), the image formation model is a time-lapse version of Eq. 3, which can be expressed as:

(4) f(t)=F(d)h(d,t)+σ(t),

where f(t) is a time-lapse sinogram constructed by the projection measurements at the time bin t of a TCSPC temporal histogram.

For spectral FLIM measurement using a gated ultrafast camera (Fig. 1c), the image forward model is a function of both time t and wavelength λ:

(5) f(t,λ)=F(d)h(d,t,λ)+σ(t,λ),

where f(t,λ) is a spectrally resolved, time-lapse sinogram constructed by the projection measurements at the gated time t and wavelength λ.

The image reconstruction of LIFT-FLIM and -sFLIM involves solving the inverse problems of Eq. 4 and 5, respectively. Like standard computed tomography, this can be accomplished through simple inverse Radon transform or more advanced optimization algorithms like a Fast Iterative Shrinkage-Thresholding Algorithm (FISTA) [27,28]. We depict the workflow for processing the light field data, such as image refocusing, extending the depth of field, and rendering a 3D image in Methods.

**Fig. 2.**
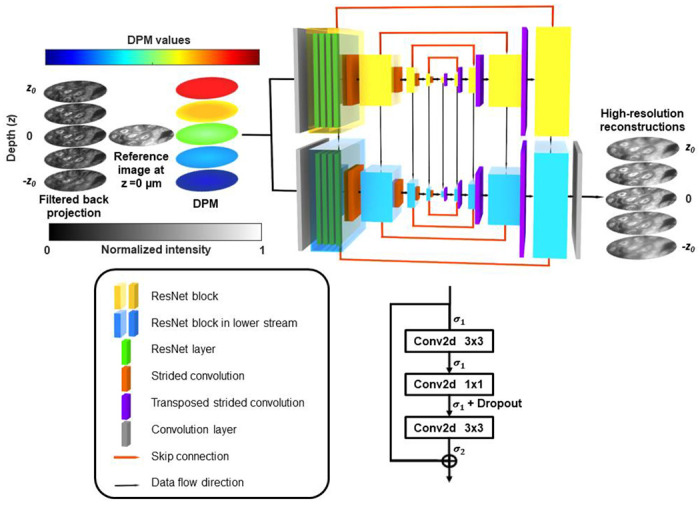
Deep-learning-based image enhancement neural network. The network consists of two down- and up-sampling streams. Each stream has five ResNet blocks in both down-sampling and up-sampling paths. Each ResNet block contains four ResNet layers, and each ResNet layer has two 3×3 convolutional layers and one 1×1 convolutional layer, as indicated in the bottom right panel. Strided convolutional layers were added between the two adjacent ResNet blocks to halve the spatial dimensions in the down-sampling path, and conversely transposed strided convolutional layers were utilized to implement up-sampling in the up-sampling path. The spatial dimensions of the ResNet blocks in the sampling streams from left to right are 256×256,128×128,64×64,32×32,16×16,32×32,64×64,128×128,256×256. The central 16×16 ResNet blocks are shared by the down- and up-sampling streams. Skip connections connect each ResNet block in the down-sampling path with its counterpart block in the up-sampling path. The inputs to the network include LIFT refocused depth image stack using filtered back projection from depth $-z_{0}$ to depth $z_{0}$, reference image captured at depth zero, and a DPM stack. The output is a high-resolution image stack at the corresponding depths. DPM: digital propagation matrix. $\sigma_{1},\sigma_{2}$: activation functions. Conv2d: convolution 2D.

Akin to conventional light field cameras, LIFT-FLIM and -sFLIM divide the aperture to extract the depth information. Therefore, they have a reduced lateral resolution (∼1.8μm) compared with the native diffraction-limited resolution of the objective lens (Fig. S2). To improve the quality of the reconstructed images, we developed a deep-learning-based image enhancement neural network [29–31] (Fig. 2). The input to the neural network consists of reconstructed LIFT depth images, a diffraction-limited reference image captured at the depth zero, and digital propagation matrices (DPMs), which represent the axial distance from the reference image plane to the target plane on a per-pixel basis [32]. The neural network then uses a PixelCNN++ architecture [33] to generate high-resolution outputs at corresponding depths. Further details about the network can be found in Methods. The axial resolution, determined by the NA of the objective lens and the number of views acquired, was measured to be ∼3.0μm for point objects (Fig. S2). The temporal resolutions of LIFT-FLIM and -sFLIM depend on the characteristics of the image sensor. For example, using a linear SPAD array provides a temporal resolution of 50ps with TCSPC [34], whereas using a gated ultrafast camera yields a temporal resolution of 70 ps [35].

### LIFT-FLIM of mixed fluorescent beads

**Fig. 3.**
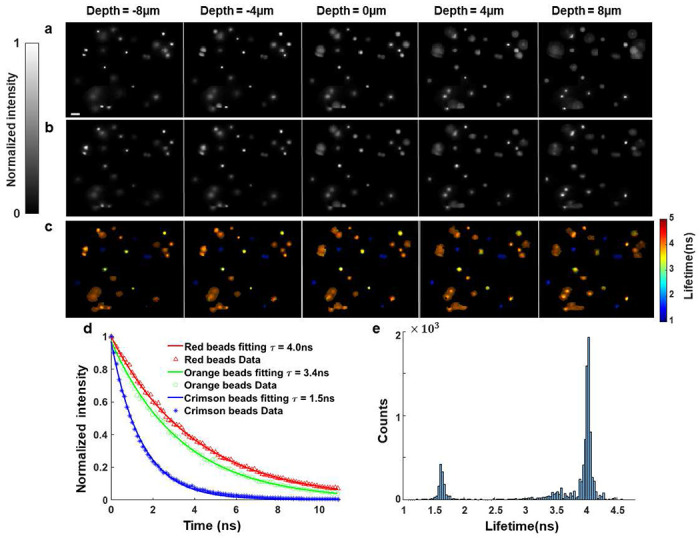
LIFT-FLIM of mixed fluorescent beads. **a.** Reference intensity images at depths of -8μm,-4μm,0μm,4μm, and 8μm. **b.** Time-integrated LIFT-FLIM images at the corresponding depths. The refocusing to continuous depths is visualized in Movie 1. **c.** Lifetime images at the corresponding depths. **d.** Fluorescence decay curves at representative beads’ locations. **e.** Histogram of pixel lifetimes at depth zero. Scale bar: 20μm.

We validated the 3D lifetime imaging performance of LIFT-FLIM on fluorescent beads. We mixed three types of fluorescent beads with lifetimes of 1.5ns,3.4ns, and 4.0ns, respectively, in an agarose gel. We simultaneously excited the beads using a filtered supercontinuum laser and imaged the fluorescence using LIFT-FLIM with a linear SPAD array. Moreover, we captured the ground-truth intensity images (Fig. 3a) at depths from −8μm to 8μm using a reference camera by mechanically scanning the microscope’s focus.

To compare LIFT-FLIM images with the ground-truth images obtained, we summed the signals at all time bins in the TCSPC temporal histogram and reconstructed the time-integrated images at the corresponding depths (Fig. 3b). The resulting images exhibit a high degree of similarity to the ground-truth images, demonstrating the system’s numerical refocusing ability. We further generated the time-lapse LIFT-FLIM images and computed the average lifetime at each image pixel using mono-exponential curve fitting (Fig. 3c). Three representative fluorescence decays reconstructed at beads’ locations are shown in Fig. 3d. The derived fluorescence lifetimes are consistent with the beads’ specifications. Furthermore, we generated a histogram of the lifetimes of all pixels at depth zero (Fig. 3e). This histogram displays three distinct peaks that correspond to the lifetimes of three different types of fluorescence beads. This observation reinforces the reliability and accuracy of our lifetime measurement.

### LIFT-FLIM of a mouse kidney tissue section

**Fig. 4.**
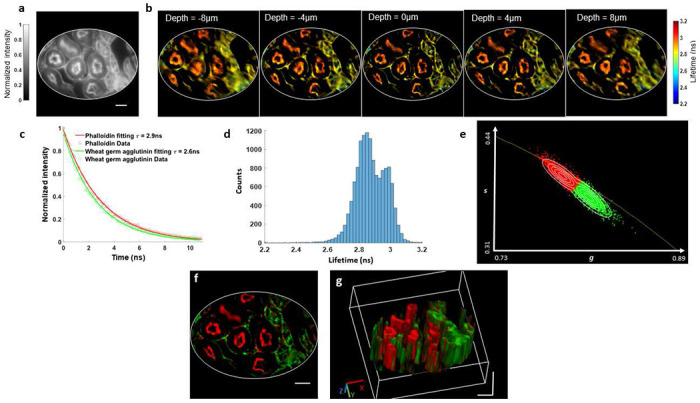
LIFT-FLIM of a mouse kidney tissue section. **a.** Reference intensity image at depth zero. **b.** Reconstructed lifetime images at depths of -8μm,-4μm,0μm,4μm, and 8μm. The refocusing to continuous depths is visualized in Movie 2. **c.** Fluorescence decay curves at two representative fluorophore locations. **d.** Histogram of pixel lifetimes at depth zero. **e.** Phasor plot. The data points were pseudocolored based on its probability belonging to a specific cluster (Red, Phalloidin; Green, WGA). The probability contour lines ranging from outer to inner space correspond to values of 0.1,0.3,0.5,0.7, and 0.9. **f.** Unmixed fluorophore image at depth zero. Red channel, Phalloidin. Green channel, WGA. **g.** 3D visualization of unmixed fluorophores’ distribution. Visualization from other perspective angles is provided in Movie 3. Scale bars in all figures: 20μm.

We tested LIFT-FLIM on a standard biological sample (a mouse kidney section, FluoCells ${\;}^{M}$ Prepared Slide from ThermoFisher) and demonstrated its ability in lifetime unmixing. The sample was stained with Alexa Fluor 488 wheat germ agglutinin (WGA) for labeling cell membrane and Alexa Fluor 568 phalloidin for labeling filamentous actin (F-actin). These two fluorophores have distinct but close fluorescence lifetimes (Alexa Fluor 488, 2.6ns *vs*. Alexa Fluor 568, 2.9ns). The fluorescence intensity image captured at the focal plane by the reference camera is shown in Fig. 4a. The refocused LIFT-FLIM fluorescence lifetime images at representative depths are displayed in Fig. 4b. Figure 4c shows the fluorescence decay curves measured at two fluorophore locations, and Fig. 4d shows the histogram of all pixels’ lifetimes at depth zero, where the two peaks indicate the two underlying lifetime components.

Next, we applied an unsupervised phasor approach [36,37] to the fluorescence lifetime data and calculated the probability of each pixel belonging to a specific lifetime component cluster. Figure 4e displays the phasor plot for the fluorescence lifetime image at depth zero, with each data point color-coded to represent its corresponding probability and overlaid with probability contour lines. We then classified the image pixels in the time-integrated LIFT-FLIM image based on this probability and unmixed the fluorophores into pseudo-colored channels. Figure 4f shows a representative unmixed image at depth zero (red channel, phalloidin; green channel, WGA). Repeating this procedure for all depths yields a 3D unmixed image, as shown in Fig. 4g.

### LIFT-FLIM of a human lung cancer pathology slide

We demonstrated LIFT-FLIM in autofluorescence imaging of an unstained human lung cancer pathology slide. Previous studies show that FLIM can access tumor metabolism by imaging endogenous chromophores such as NAD(P)H and FAD, enabling its application in cancer diagnosis and intraoperative surgical guidance [38,39]. Particularly in pathological imaging, FLIM holds great promise as an alternative approach for label-free detection of tissue lesions [8,40,41]. However, conventional FLIM microscopes with a high collecting NA suffer from a shallow depth of field. When imaging a panoramic FOV through multiple captures and stitching, the system must mechanically adjust its focus at each position to correct for potential focal drift that can occur during extensive scanning, complicating the imaging procedure. Here we show that, by using numerical refocusing, LIFT-FLIM enables an extended depth of field and allows for capturing an all-in-focus image without the need for accounting for the focal drift.

**Fig. 5.**
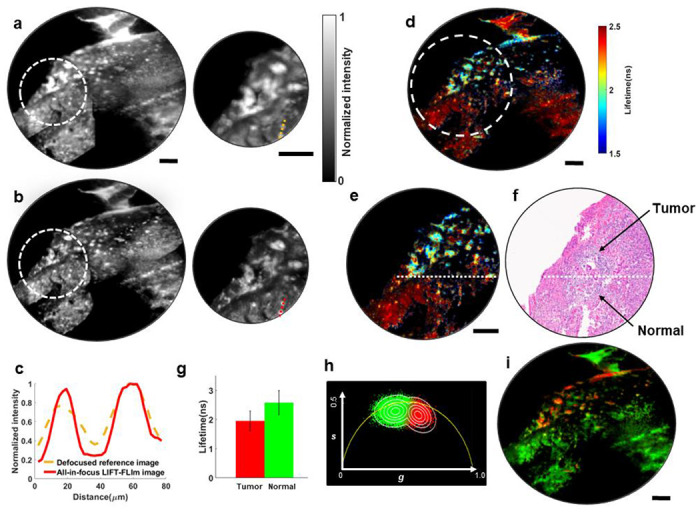
LIFT-FLIM of a human lung cancer pathology slide. **a.** Left panel: stitched reference intensity image. Right panel: zoom-in image of the circled area in the left panel. The image is blurred due to focal drift during extensive scanning. **b.** Left panel: stitched all-in-focus time-integrated LIFT-FLIm image. Right panel: zoom-in image of the circled area in the left panel. **c.** Intensity profiles of dashed lines in a and b. **d.** Stitched all-in-focus lifetime image. The lifetime image is masked with an intensity threshold **e.** Zoom-in image of the circled area in **d**. **f.** Hematoxylin and eosin (H&E) stained image from an adjacent tissue slice. The tumor/normal tissue boundary was identified by a pathologist and annotated with a white dashed line. **g.** Average pixel lifetimes in the tumor and normal tissues areas in **e.** The standard deviation (SD) is shown as error bars. **h.** Phasor plot. The data points were pseudocolored based on its probability belonging to a specific cluster (Red, tumor; Green, normal). The probability contour lines ranging from outer to inner space correspond to values of 0.1,0.3,0.5,0.7, and 0.9. **i.** Classified tissue map. Red channel, tumor; Green channel, normal. Scale bars in all figures: 100μm.

We excited the sample at 450nm and collected the autofluorescence in the range of 490-700nm. The primary endogenous fluorophore that accounts for the fluorescence emission at this wavelength is flavin adenine dinucleotide (FAD). To image a large FOV, we scanned the sample and stitched the images. The resultant fluorescence intensity image captured by the reference camera is shown in Fig. 5a, where certain parts of the FOV are blurred due to the focal drift. In contrast, LIFT-FLIM can numerically correct for this defocus error in post-processing and form an all-in-focus image, as shown in Fig. 5b. For quantitative comparison, we plotted signal intensities along a dashed line in Fig. 5a–b and show the results in Fig. 5c. The image features appear to have much sharper edges in LIFT-FLIM compared to those captured by the reference camera (∼36% reduction in full-width at half maximum). Next, we computed the lifetimes for the stitched all-in-focus LIFT image and presented a lifetime map in Fig. 5d. A zoom-in area (Fig. 5e) shows a significant level of lifetime heterogeneity. To correlate this observation to the tissue state, we stained an adjacent slide from the same tissue sample using standard hematoxylin and eosin (H&E) and imaged it under a widefield microscope. After the histological image was obtained, a pathologist reviewed it to identify the boundary between the tumor and normal tissue, as illustrated in Fig. 5f. Comparing the average pixel lifetimes above (1.9±0.3ns) and below (2.6±0.4ns) the annotated boundary (Fig. 5f) reveals a significant difference (Fig. 5g). The observed reduction in autofluorescence lifetimes in the tumor areas compared to that in the normal tissue is consistent with previous reports [42–44] and may indicate a shift towards glycolysis and cancer metabolism [45]. To classify the tissue based on the lifetime, we again applied an unsupervised phasor approach [36,37] to the fluorescence lifetime data. The resultant phasor plot and classified tissue map are shown in Fig. 5h and 5i, respectively (red channel, tumor; green channel, normal tissue).

### LIFT-sFLIM of lung organoids

We demonstrated LIFT-sFLIM in 3D multiplex imaging of lung organoids. Organoids, 3D multicellular stem-cell-derived constructs that mimic *in vivo* tissue, have gained growing interest for modeling tissue development and disease [46–48]. Particularly, organoids hold great promise for high-content phenotypic screening because they recapitulate many aspects of parent tissues and can be derived from patient material, rendering them ideal model systems for personalized medicine and drug discovery [49–53].

One primary challenge for high-content phenotypic screening of organoids is extraction of multivariate information from organoids labeled with multiple biomarkers [54–56]. Here we show that, by acquiring both the spectral and lifetime information, LIFT-sFLIM provides a powerful solution to overcome this challenge. We cultured lung alveolar organoids with different combinations of primary healthy human lung fibroblasts and epithelial cells grown on alginate scaffolds that mimic the alveolar micro-architecture [57]. We used the antibodies tabulated in **Supplementary Table 1** and labeled epithelialmesenchymal transition by α smooth muscle actin ( α-sma) expression, ECM deposition by collagen (collagen I) expression, cell apoptosis by SMAD signaling pathway (smad3), and cellular senescence by P16^INK4A^ (p16) expression.

**Fig. 6.**
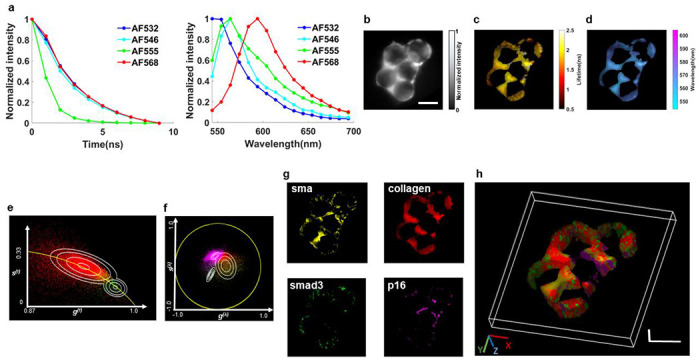
LIFT-FLIM of lung organoids. **a.** Fluorescence decay curves (left panel) and emission spectra (right panel) of the fluorophores used. **b-d.** Reconstructed LIFT-sFLIM (**b**) intensity image, (**c**) wavelength-integrated lifetime image, and (**d**) time-integrated wavelength image at depth zero. **e.** Lifetime phasor plot. The data points were pseudocolored based on its probability belonging to a specific cluster (Red, sma, collagen, p16; Green: smad3). The probability contour lines ranging from outer to inner space correspond to values of 0.2,0.35,0.65, and 0.95. **f.** Spectral phasor plot. The data points were pseudocolored based on its probability belonging to a specific cluster (Magenta: collagen, smad3; Yellow: sma; Green, p16). The probability contour lines ranging from outer to inner space in the magenta and yellow clusters correspond to values of 0.45, 0.65, and 0.85, while the contour lines in the green cluster correspond to values of 0.25, 0.45,0.65, and 0.85. **g.** Unmixed component images at depth zero. **h.** 3D visualization of unmixed fluorophores’ distribution in the organoid. Visualization from other perspective angles is provided in Movie 4. Scale bar: 100μm in all figures.

Figure 6a depicts the fluorescence emission decay curves and spectra of the four fluorophores that were utilized in the secondary antibodies. While the fluorophores AF 532, 546, and 568 have close fluorescence lifetimes, their spectral emission peaks are well separated. On the other hand, AF 546 and AF 555 exhibit significant spectral overlaps but differ in fluorescence lifetimes. The combination of four fluorophores used in this study presents a challenge for conventional imaging techniques. Specifically, neither FLIM nor spectral imaging alone can simultaneously capture and distinguish all four fluorophores. This limitation underscores the need for innovative imaging approaches, such as LIFT-sFLIM, which can integrate both spectral and temporal information to enable reliable separation and quantification of multiple fluorophores in complex biological samples.

Using LIFT-sFLIM, we acquired a five-dimensional (5D) dataset (x,y,z,t,λ)(x,y,z, spatial coordinates; t, fluorescence decay time; λ, wavelength). Figure 6b shows the LIFT-sFLIM reconstructed intensity image at depth zero. The wavelength-integrated lifetime image and time-integrated wavelength image at depth zero are shown in Fig. 6c–d, respectively. To unmix the fluorophores, we applied a spectral-lifetime phasor approach to the 5D dataset. The resultant color-coded phasor plots in the lifetime and spectral domains are shown in Fig. 6e–f, respectively. Consistent with the spectral and lifetime data presented in Fig. 6a, our analysis revealed two distinct clusters in the lifetime phasor plot and three distinct clusters in the spectral phasor plot. By combining spectral and temporal information, we separated the fluorophores into four color-coded channels. Representative images at depth zero are shown in Fig. 6g. By repeating this procedure at all depths, we generated a 3D color-coded image that depicts the distribution of each fluorophore in the organoid, as shown in Fig. 6h.

## Discussion

Using LIFT-FLIM for 3D lifetime imaging offers a crucial benefit of reducing the number of scanning steps required compared to traditional point- or line-scanning time-domain FLIM techniques. To produce a 3D image of $N_{x}\times N_{y}\times N_{z}$ voxels, a FLIM system that uses point- or line-scanning requires a total of $N_{x}\times N_{y}\times N_{z}$ or $N_{y}\times N_{z}$ (if line scans are done along the y axis) scanning steps, respectively. Here, $N_{x},N_{y}$, and $N_{z}$ denote the number of spatial samplings in a 3D space. For simplicity, we consider $N_{x}=N_{y}=N$. In contrast, because LIFT-FLIM distributes projection measurements into different views, it demands only $N_{\theta }$ scanning steps, where $N_{\theta }$ is a total number of projection angles. Therefore, LIFT-FLIM reduces the scanning steps required by a factor of $N^{2}\times N_{z}/N_{\theta }$ or $N\times N_{z}/N_{\theta }$ compared to point- or line-scanning systems.

For non-compressive measurement, we set $N_{\theta }$ equal to N. Additionally, as demonstrated in Supplementary Materials, our findings indicate that, in the light field imaging, the effective number of depth samplings, $N_{z}$, equals the number of angular samplings, K. As a result, the scanning reduction factor is either N×K or K when compared to point- or line-scanning systems. With our current N and K values set at 180 and 15, respectively, the resulting scanning reduction factors are 2,700 and 15 in comparison to point- or line-scanning systems.

Alternatively, like sparse-view computed tomography [58], we can choose an $N_{\theta }$ less than N for compressive measurement. We define a compression ratio (CR) as

(6) $$\text{CR}=N/N_{\theta }.$$

To quantify the dependence of the reconstructed image quality on the CR, we adopted the peak signal-to-noise ratio (PSNR) and structural similarity index measure (SSIM) as evaluation metrics. We varied the CR by increasing $N_{\theta }$ and calculated the corresponding PSNRs and SSIMs. Figure S3 illustrates reconstructions of sparse and complex objects at different CR values. In general, reducing the CR improves both PSNR and SSIM in the reconstructed image. Additionally, our findings suggest that the quality of the reconstructed image is highly dependent on the CR for complex objects, like the lung tumor image displayed in Fig. S3c. In such cases, a lower CR value, less than 4.5, is necessary to achieve high-quality image reconstruction (SSIM ⩾0.9). Conversely, when imaging a sparse object, such as an USAF resolution target, a CR of 9 is sufficient to recover a high-quality image. Therefore, by adjusting $N_{\theta }$, LIFT-FLIM can tailor the CR to the complexity of a sample, resulting in effective measurements for a given object.

The imaging speed of LIFT-FLIM is determined by the total number of projections $N_{\theta }$ acquired and the time duration at each projection. For LIFT-FLIM using a linear SPAD array, the duration at each projection includes both the pixel exposure time and temporal histogram readout time. For LIFT-sFLIM using a gated ultrafast camera, the duration at each projection equals the product of the number of time gates and the camera frame time. Importantly, when imaging simple objects, the system can be operated in the compressive measurement mode, where a reduced $N_{\theta }$ can be acquired to accelerate the imaging speed without compromising the image quality.

The spatial resolution of LIFT-FLIM is fundamentally limited by optical diffraction when performing non-compressive measurements. Due to the division of the aperture, LIFT-FLIM has a lateral resolution of λf/D, where λ is the wavelength, f is the focal length of the objective lens, and D is the sub-aperture diameter associated with a perspective image. Given K views, $D=D_{0}/\sqrt{K}$, where $D_{0}$ is the original aperture of the objective lens. Therefore, the lateral resolution is $\sqrt{K}$ times greater than the native resolution of the objective lens. Although this is a common issue encountered by all light field cameras, we can mitigate it by acquiring fewer views and increasing the sub-aperture size to enhance the resolution at the expense of reduced depth accuracy. On the other hand, when performing compressive measurements, the spatial resolution of LIFT-FLIM is practically limited by the CR. While a higher CR is favored in terms of imaging speed, it deteriorates the reconstructed image quality and resolution for complex objects. Hence, selecting an appropriate CR value for a given object involves striking a balance between imaging speed and resolution.

LIFT-FLIM images can be reconstructed and analyzed in real-time. For instance, when processing uncompressed measurement data, a simple inverse Radon transform takes about 0.13 seconds per time bin on an Nvidia RTX3080Ti GPU with CUDA. Subsequently, deep learning enhancement and phasor analysis require 0.079 and 0.024 seconds, respectively. Parallel computing reduces the total post-processing time to less than 0.3 seconds.

The light throughout of LIFT-FLIM depends on the sub-aperture size of a perspective image, the ratio of projection line image width to the detector pixel’s size, and the fill factor of the image sensor. As detailed in Supplementary Materials, the LIFT-FLIM is built on an unfocused light field imaging configuration, where the projection line width at the image sensor equals to the sub-aperture diameter, D, multiplying with a pupil demagnification ratio, r. Given the pixel pitch, p, and fill factor, κ, the percentage of light measured by the image sensor pixel is

(7) $$\zeta_{\text{FILM}}=\frac{D}{D_{0}}\times \frac{p\kappa }{Dr}=\frac{p\kappa }{D_{0}r}.$$

Here $D/D_{0}$ describes the light loss due to the view selection during pupil scanning, where $D_{0}$ is the original aperture of the objective lens. Therefore, a lower pupil demagnification ratio (*i.e.*, a shorter focal length of the cylindrical lens in Fig. 1a) can lead to a higher system light throughput. In our current system, due to use of only off-the-shelf optics and a SPAD array, we have r equal to 0.1, resulting in an overall light throughput of 0.01. To further enhance the system performance, one possible approach is to utilize custom optics that feature a lower pupil demagnification ratio, r, together with a rectangularly shaped SPAD pixel that has a longer pixel pitch, p, in the direction of the projection line width. Alternatively, instead of scanning the pupil to choose the views, it is possible to simultaneously capture all perspective images by employing an array of dove prisms with different orientations, as we have previously demonstrated [26]. However, this setup necessitates the use of multiple linear SPAD arrays, each of which measures a projection line image in a synchronized fashion.

On the other hand, for LIFT-sFLIM using a gated ultrafast camera, the light throughput is determined by the sub-aperture size of a perspective image, the diffraction efficiency of the grating, χ, and quantum efficiency of the gated ultrafast camera, η.

(8) $$\zeta_{\text{sFILM}}=\frac{D}{D_{0}}\times \chi \times \eta .$$

Since $D/D_{0}=1/\sqrt{K}$, where K is the total number of views acquired, Eq. 8 can be rewritten as $\zeta_{\text{sFILM}}=\chi \eta /\sqrt{K}$. Hence, reducing the number of angular samplings can boost the light throughput, but this comes at the cost of decreased depth accuracy. Noteworthily, here the pupil magnification ratio, r, has no effect on the light throughput. Rather, it governs the spectral resolution of the system like in a conventional pushbroom imaging spectrometer [59,60].

To investigate how the number of photons received at a pixel affects the quality of the reconstructed image, we conducted simulations under a shot-noise-limited condition. Provided that the pixel with the maximum count in the image collects M photons, the corresponding shot noise is $\sqrt{M}$ photons. We introduced photon noise to all pixels in the projection images and reconstructed the images with various values of M, while maintaining a constant number of projections across all data points in the plot. Figure S4 presents the reconstruction results of a Shepp-Logan phantom under different M values. The results indicate that a larger M (*i.e.*, more photons) can lead to a higher PSNR. For high-quality image reconstruction (PSNR ⩾20dB),M must be greater than 64 photons.

To sum up, we have created a highly data-efficient 3D FLIM technique that relies on light field tomography and extended its capabilities to 3D sFLIM. We believe that LIFT-FLIM and -sFLIM will find broad applications in high-throughput and high-content imaging of biological cells and tissues, opening up new avenues for both fundamental and translational biomedical research.

## Methods

### Experimental setup

In a LIFT-FLIM and -sFLIM system, we used an epi-fluorescence microscope (IX83, Olympus) as the front-end optics and excited the sample with a pulsed laser source (SuperK FIANIUM, FIU-15, NKT Photonics, for LIFT-FLIM; SIRIUS GR-2, Spark Laser, for LIFT-sFLIM). The emitted fluorescence is collected by a microscope objective lens (UPLXAPO60XO, Olympus; UPLXAPO20X, Olympus), and an intermediate fluorescence image is formed at the side image port of the microscope.

To split the light, we employed a beam splitter (BSX16, Thorlabs), which transmits 10% of the light to a reference camera (CS2100M-USB, Thorlabs) and reflects 90% of the light to the LIFT-FLIM camera. We placed a scanning mirror (MR-10-30, Optotune) at the intermediate image plane to shift the pupil image.

The fluorescence is then directed through a 4f system, which consists of two lenses (ACT508-250-A and AC254-150-A, Thorlabs) with a focal length of 250mm and 150mm, respectively. To rotate the perspective image, we mounted a Dove prism (PS990M, Thorlabs) on a motorized rotation stage (PRM1Z8, Thorlabs) and positioned the assembly at the Fourier plane of the 4f system. We also positioned a cylindrical lens (LJ1095L1-A, Thorlabs, invariant axis along the y-axis) 131mm after the second lens in the 4f system, which generates a 1D en-face projection of a perspective image along the y-axis. To locate the projection line image, we identified the line with the smallest width. Additionally, we compensated for the focal shift and spherical aberration introduced by the cylindrical lens by defocusing.

The subsequent system is split into two arms, namely the LIFT-FLIM and LIFT-sFLIM arms. The former employs a linear SPAD array [34], while the latter utilizes a 2D ultrafast time-gated camera (High rate image intensifier, LaVision). To switch the light path between the two arms, we placed a flip mirror (TRF90, Thorlabs; PF10-03-G01, Thorlabs) at the line image plane.

When the mirror is positioned at 1, the fluorescence is directed towards the LIFT-FLIM sub-system through a camera lens (YN100mm F2, YONGNUO) and directly measured by the linear SPAD camera. The linear SPAD camera comprises 256 effective CMOS SPAD pixels with a pitch of 26.2μm. Operating in the TCPSC mode, the SPAD camera provides a temporal resolution of 50 picoseconds [34]. It is connected to a FPGA (Spartan 6, Xilinx) with 64 time-to-digital converters (TDCs) and histogram engines, enabling it to process up to 8.5 giga-photons per second. By rotating the dove prisms in a set of angles at assigned views, we sequentially acquired the 1D en-face projections and constructed a sinogram.

In position 2 of the flip mirror, the emitted fluorescence is directed to the LIFT-sFLIM sub-system. The line image is relayed to the image sensor plane by a pair of camera lenses (YN100mm F2, YONGNUO). To disperse the line image along the x-axis, we positioned a transmission diffraction grating (GT50-03, Thorlabs) at the Fourier plane of the relay system. The resultant dispersed projection image is then sampled in time by an ultrafast time gate and further relayed to a 2D camera (CS2100M-USB, Thorlabs) by a camera lens (YN100mm F2, YONGNUO). By varying the delay between the time gate and the laser reference signal, we acquired a series of time-resolved dispersed projection images. To synchronize the scanning mirror, the dove prism rotation stage, the camera, and the laser, we employed a digital delay generator (DG645, Stanford Research Systems). To maximize information content for image reconstruction, we chose the dove prism rotation angles from a set of angles that are evenly spaced in the range of $\left[0,90^{\circ }\right]$.

To tune the illumination wavelength from the supercontinuum laser, we built a wavelength-selecting module (Fig. S5) using a digital micromirror device (DMD). The collimated white laser beam is first dispersed by a transmission diffraction grating (GT50-03, Thorlabs) and line focused onto the surface of the DMD (DLP LightCrafter 6500, Texas Instruments) through a cylindrical lens (LJ1125L1-A, Thorlabs). The broadband illumination has a line dispersion of 54.4nm/mm on the DMD surface. The DMD has 1920×1080 micromirrors, each of which can be individually tilted $\pm 12^{\circ }$ relative to the norm. Each column of the DMD corresponds to a different wavelength with a 0.4nm/column wavelength resolution. By adjusting the mirror pattern, we can select any desired illumination wavelengths. The laser light of selected wavelengths is then spatially recombined by another identical set of cylindrical lens and diffraction grating and directed towards the LIFT-FLIM sub-system.

When imaging the mixed fluorescence beads and mouse kidney tissue section, we used multiband excitation with two different wavelengths (488 nm and 561nm) and separated fluorescence from excitation using the combination of a multiband dichroic mirror (ZT405/488/551/647rpc, Chroma) and a multiband emission filter (ZET405/488/561/647m, Chroma). For imaging the human lung cancer pathology slide, we used 450nm laser excitation, a 495nm dichroic mirror (T495lpxr, Chroma), and a long-pass emission filter (ET500lp, Chroma). In the case of lung organoids, we used 532 nm laser excitation, a 532nm dichroic mirror (ZT532rdc, Chroma), and a long pass emission filter (ET542lp, Chroma). The laser fluence at the sample focal plane was approximately $9.9\times 10^{-7}\;J/{cm}^{2},1.1\times 10^{-7}\;J/{cm}^{2}$, and $2.9\times 10^{-3}\;J/{cm}^{2}$ for the mouse kidney section, lung cancer pathology slide, and lung organoid imaging experiments, respectively. These laser fluences were well below the cell damage threshold of $4\;J/{cm}^{2}$ [61,62].

### Image reconstruction

To obtain an image of a monochromatic scene at a specific time point and depth from the measurement described by Eq. 3, we iteratively solve an optimization problem:

(9) $$\text{argmin}\;∥\text{f}-F(d)h(d){∥}_{2}^{2}+\mu ∥\varphi (h(d){∥}_{1},$$

where $∥\cdot {∥}_{2}$ denotes the $l_{2}$ norm, $∥\cdot {∥}_{1}$ denotes the $l_{1}$ norm, and φ(⋅) is a data regularization term. μ is a hyperparameter that balances the data fidelity and regularization term. In the framework of regularization by denoising [63], φ(⋅) is not explicitly specified, and the regularization can be implemented by a state-of-the-art image denoising algorithm such as BM3D or a neural network. We adopted the BM3D and total variation (TV) denoisers for the regularization due to the availability of efficient algorithms [64]. Besides the iterative method [27,28], inverse Radon transformation is an alternative approach that could have been used for image reconstruction with lower computational cost.

### Refocusing and extending the depth of field

A light field acquired by conventional light field cameras can be parameterized by the aperture plane (u,v) and the image plane (x,y). Indexing view k as $\left(u_{k},v_{k}\right)$, the image $P_{k}(x,y)$ observed from view k can be related to a reference image feature kernel h(x,y) by

(10) $$p_{k}(x,y)=h\left(x-su_{k},y-sv_{k}\right),$$

where s is a depth-dependent shearing parameter (Supplementary Note 1). In conventional light field imaging, refocusing is performed by shifting and adding the subaperture images [65]. Unlike conventional light field cameras, LIFT-FLIM first rotates a perspective image, followed by transforming the rotated image into a line. Therefore, the depth-dependent shearing must be performed parallel to the projection axis.

For sub-aperture $\left(u_{k},v_{k}\right)$ at the projection angle of θ, the shearing of 1D sub-aperture projection is given by

(11) $$s\cdot u_{k}\cdot \text{sin}\;\theta -s\cdot v_{k}\cdot \text{cos}\;\theta .$$

For numerical refocusing, we applied the correspondent shearing factor to each projection image and updated sinogram for reconstructing the depth image (Supplementary Note 1).

Extending the depth of field can be achieved through a similar approach to conventional light field imaging, which involves refocusing onto different depths, extracting the sharpest feature for each pixel, and assembling an all-in-focus image [66].

### System calibration and resolution

#### Scanning mirror calibration

To ensure that the scanning range of sub-apertures fully utilizes the entire aperture of the objective lens, we calibrated the scanning mirror’s horizontal and vertical tilt angles. Additionally, to optimize the light throughput of each sub-aperture, we employed a rectangular iris instead of a round one at the aperture stop. The rectangular shape reduces the gaps between adjacent scanned pupil positions and allows more light to pass through a sub-aperture.

#### Projection center calibration

To calibrate the central position of each projection line image at the sensor plane, we imaged a pinhole (P10D, Thorlabs) positioned at the center of the FOV on the sample stage. We captured images of the pinhole at every projection angle θ and view k and directly localized the center of each line image as $y_{p0}^{\left(\theta ,k\right)}$ (Supplementary Note 1). Subsequently, we extracted the projection data based on the center location to form a sinogram.

#### Spectrum calibration and resolution

To calibrate the spectral response, we positioned a pinhole (P10D, Thorlabs) at the sample stage and illuminated it with monochromatic light at varied wavelengths. The resulting pixel locations of the projections were recorded and fitted with a linear polynomial, as illustrated in Fig. S7a. The slope of the line determines the spectral sampling of the system, which was calculated to be 0.14nm. The spectral resolution is defined as the full-width at half maximum (FWHM) of the spectral response. A 1nm bandpass filter (FL532-1, Thorlabs) was used to limit the source wavelength for this measurement, and the raw spectral response is displayed in Fig. S7b, where the FWHM was approximately 9.2nm. However, this width was a convolution of the geometrical image of the pinhole on the camera (approximately 7 pixels), the bandwidth of the light source (approximately 7 pixels), and the system spectral resolution. The width of a convoluted function (in pixels) can be computed as [67]:

(12) $$w\left(f_{1}\text{*}f_{2}\text{*}f_{3}\right)=w\left(f_{1}\right)+w\left(f_{2}\right)+w\left(f_{3}\right)-2,$$

where w denotes the width of the function, * denotes the convolution operator, and $f_{i}(i=$ 1,2,3) denotes the individual function in a discrete form. Based on this equation, the width of the spectral resolution on the camera was estimated to be 48 pixels. Given a 0.14nm spectral sampling, the spectral resolution is 6.6nm.

#### Spatial resolution and field-of-view(FOV)

To quantify the spatial resolution, we imaged a fluorescence bead with a diameter of 4 μm (F8858, Thermo Fisher), and the raw reconstruction results are presented in Fig. S2a-d, where the lateral and axial full-width at half maximum (FWHM) are approximately 4.6μm and 5.7μm, respectively. However, the lateral FWHM width was a convolution of the geometric image of the bead on the camera (around 4 pixels) and the system lateral resolution. The width of a convoluted function can be calculated as [67]:

(13) $$w\left(f_{1}\text{*}f_{2}\right)=w\left(f_{1}\right)+w\left(f_{2}\right)-1$$

where w denotes the width of the function, * denotes the convolution operator, and $f_{i}(i=$ 1,2) denotes the individual function in a discrete form. Using this formula, the width of the lateral resolution on the camera was calculated to be 2 pixels. Given a 0.9μm spatial sampling (camera pixel pitch of 26.2μm divided by system magnification ratio of 29), the lateral resolution was estimated to be 1.8μm. Similarly, for axial FWHM, the width was a convolution of the bead size and the system lateral resolution. As a result, the axial resolution was estimated to be 3μm.

We also imaged a group of bars from a USAF resolution target (Group 7 element 3-6) along both horizontal and vertical directions and plotted the intensities along the dashed line. The image visibility, defined as $\left(I_{max}-I_{min}\right)/\left(I_{max}+I_{min}\right)$, where I is the intensity, was calculated for each group of bars using the peaks and valleys of the intensity. With a visibility threshold of 0.2, the spatial resolution of the bars was determined to be 2.2μm along both vertical and horizontal directions, indicating an isotropic resolution. The FOV of our system was measured to be 227μm×143μm when using a 60x microscope objective lens (UPLXAPO60XO, Olympus).

#### Depth calibration

To calibrate the depth, we utilized a fluorescence bead (C16509, Thermo Fisher) and translated it along the depth axis from -16μm to 16μm with a 2μm step. At each depth, we captured an image and then performed digital refocusing by adjusting the shearing parameter, as described in Methods: Refocusing and extending the depth of field. The goal was to identify the shearing parameter that would bring each image into the sharpest focus, which was determined by maximizing a focus measure (*e.g.*, sum of modified Laplacian) for each pixel in the image [21]. The best focus shearing parameter at each physical depth was then recorded. The resultant shearing parameter to depth curve was fitted with linear models. With this calibration curve, we can digitally refocus a 3D object to a specific depth using the correspondent shearing parameter. To validate the accuracy of our shearing parameters, we imaged 3D fluorescence beads in an agarose gel and compared LIFT refocusing against the ground-truth depth images captured by a reference camera, as shown in Fig. 3.

#### Camera registration

To register the LIFT-FLIM and -sFLIM image with the reference camera, we imaged a 7 by 13 grid pattern. We then extracted the point locations from the reconstructed LIFT-FLIM and -sFLIM images and the reference image and calculated the homography matrix to establish a pixel-to-pixel correspondence between the two cameras. The reprojection error using the homography is less than one pixel, ensuring an accurate registration. This homography matrix is then used to register the LIFT-FLIM and -sFLIM images with reference images for deep learning reconstruction.

### Preparation of training data for deep learning

To generate the ground truth depth image stack, we translated the sample stage along the depth axis while capturing images using the reference camera. The resulting image stack was then transformed to the LIFT-FLIM and -sFLIM camera coordinates using a homography matrix obtained through camera registration. We subtracted a background image from the warped image stack.

The LIFT-FLIM and -sFLIM images were captured at depth =0, and a depth image stack was computed by numerical refocusing. We calculated a uniform DPM at each depth and appended them to the reconstructed image stack. We created a mask by setting a threshold to the reconstructed image to identify regions with sample fluorescence signals above the background. This mask was then applied to both the ground truth image stack and the LIFT-FLIM and -sFLIM image stack. For training, we constructed a total of 200 image stack pairs per task in the training dataset, each comprising an input image collection (LIFT-FLIM/sFLIM reconstruction stack, DPM stack, and a wide-field image at depth=0) and a ground-truth image stack.

### Deep learning network architecture and training

The PixelCNN++ architecture [33] was adopted for LIFT-FLIM and -sFLIM refocusing. As illustrated in Fig. 2, our model consists of the down- and up-sampling streams and the lower down- and up-sampling streams. Each stream has 5 ResNet blocks [68] in both the down-sampling and up-sampling paths. Each ResNet block contains 4 ResNet layers, and each ResNet layer has two 3×3 convolutional layers and one 1×1 convolutional layer. The ResNet layer utilized two activation functions $\sigma_{1},\sigma_{2}$ defined below:

$$\sigma_{1}(x)=ELU(x⊕(-x))\;\sigma_{2}\left(x_{1}⊕x_{2}\right)=x_{1}⊙\mathrm{Sigmoid}\left(x_{2}\right).$$

Here ⊕ means concatenation along the channel axis and ⊙ is element-wise multiplication. Exponential Linear Unit (ELU) [69] and Sigmoid function are defined as

$$ELU(x)=\{\begin{array}{cc} x, & \mathrm{for}\;x>0\\ \alpha (\text{exp}(x)-1), & \mathrm{for}\;x\leq 0 \end{array},$$

$$\mathrm{Sigmoid}(x)=\frac{1}{1+\text{exp}(-x)},$$

where α is a hyperparameter that controls the value to which an ELU saturates for negative net inputs. Strided convolutional layers were added between two sequential ResNet blocks to halve the spatial dimensions in the down-sampling path, and conversely transposed strided convolutional layers were utilized to implement up-sampling in the up-sampling path. Skip connections connect each ResNet block in the down-sampling path with its counterpart block in the up-sampling path such that relatively higher-frequency image features can flow through the model.

The training loss of our model is a linear combination of Fourier domain mean absolute error (FDMAE) [70,71], mean square error (MSE) and the perceptual loss:

(14) $$L(y,\hat{y})=\alpha L_{FDMAE}(y,\hat{y})+\beta L_{MSE}(y,\hat{y})+\gamma L_{p}(y,\hat{y})$$

Here α,β and γ are weights of each loss term, and were empirically set as 0.1,0.1 and 1.0, respectively. $y,\overset{ˆ}{y}\in R^{N^{2}}$ are the vectorized ground truth and predicted images, respectively. The FDMAE loss is defined as

(15) $$L_{FDMAE}(y,\hat{y})=∥Fy-F\hat{y}{∥}_{1},$$

where $F\in R^{N^{2}}$ is the Fourier transform matrix. The MSE loss is defined as

(16) $$L_{MSE}(y,\hat{y})=∥y-\hat{y}{∥}_{2}.$$

The perceptual loss is defined as the sum of MSE losses between the feature maps of y and $\overset{ˆ}{y}$ generated by a Visual Geometry Group 16 (VGG16) network [72]:

(17) $$L_{p}(y,\hat{y})=\sum_{k=1}^{K}\;w_{k}\cdot {\left\|VGG_{k}(y)-VGG_{k}(\hat{y})\right\|}_{2}$$

where $VGG_{k}(\cdot )$ represent the feature map of the input image after the $k_{th}$ block of VGG16, and $w_{k}$ is the weight for the corresponding feature maps. In this work we used the first three blocks of VGG16 for image feature extraction, i.e., K=3, and empirically set $w_{1}=$ $0.5,w_{2}=0.15,w_{3}=0.1$. An Adam optimizer with exponentially decaying learning rate was utilized for parameter optimization. The initial learning rate was set as $10^{-4}$ and the decay rate was 0.999995 per epoch.

Our models were implemented using PyTorch framework [73] on a machine with Intel Xeon W-2195 processor and four RTX 2080Ti graphic cards. All models converged after around 5000 epochs, which took approximately 2 to 3 days.

### Image stitching

We used a feature-based image stitching algorithm to create a panorama view of the human lung cancer pathology slide from multiple scanned FOVs with overlapping regions. This process involved detecting and matching image features, estimating the geometric transformation between images, and computing the transformation mapped each image onto the panorama. Moreover, to correct the artifacts in the stitched image caused by connecting the individual images, we applied an intensity averaging technique to the neighboring pixels at the artifact’s coordinates.

### Phasor Analysis

To facilitate fast and accurate analysis of LIFT-FLIM and LIFT-sFLIM data, we utilized a phasor approach to unmix the underlying chromophores. In our experiments involving mouse kidney tissue sections and human lung cancer pathology slides, we reconstructed a multidimensional array XYT at each depth Z, where X,Y and Z are the spatial dimensions, and T represents fluorescence decay time. After phasor transformation, we fed the resulting phasor coordinates into an unsupervised unmixing algorithm [36,37] to determine the probability of each pixel belonging to a specific cluster. It is important to note that the number of clusters present was assumed to be known a prior, which is generally the case as we label our samples with fluorescence probes or make assumptions regarding the sample composition [37]. For instance, we modeled our data using two clusters for the mouse kidney tissue sections (Alexa Fluor 488 WGA and Alexa Fluor 568 phalloidin) and human lung cancer pathology slide (normal and tumor). Using the probabilities obtained from the unmixing algorithm, we assigned colors to the unmixed image pixels: each cluster was assigned a unique color, and the RGB coordinates of the colors were combined using the probabilities as coefficients. This process results in a color code for each pixel, as shown in Fig. 4f, and Fig. 5i.

In LIFT-sFLIM, the image is represented by a multidimensional array *XYTS* at each depth Z, where S denotes the spectral dimension. To simplify the analysis, we focus on the T×S matrix I(t,λ) at one pixel location, where fluorescence decay is sampled at m points $\left(t=t_{1},\ldots ,t_{m}\right)$, and the spectrum is sampled at n points $\left(\lambda =\lambda_{1},\ldots ,\lambda_{n}\right)$. We also consider wavelength-integrated time decay $I^{\Lambda }(t)=\sum_{\lambda =1}^{n}\;I(t,\lambda )$ and time-integrated emission spectrum $I^{T}(\lambda )=\sum_{t=1}^{m}\;I(t,\lambda )$ to streamline the process. In our lung organoid experiment, $I^{\Lambda }(t)$ and $I^{T}(\lambda )$ can be expressed as linear combinations of four components:

(18) $$I^{\text{Λ}}(t)=f_{1}I_{1}^{\text{Λ}}(t)+f_{2}I_{2}^{\text{Λ}}(t)+f_{3}I_{3}^{\text{Λ}}(t)+f_{4}I_{4}^{\text{Λ}}(t)\;I^{\text{T}}(t)=f_{1}I_{1}^{\text{T}}(t)+f_{2}I_{2}^{\text{T}}(t)+f_{3}I_{3}^{T}(t)+f_{4}I_{4}^{T}(t),$$

where $f_{i}(i=1,2,3,4)$ are the amplitude fractions of each component with the constraint that their sum equals unity $\left(\sum_{i=1}^{4}\;f_{i}=1\right)$. Once transformed into the phasor space, $I^{\Lambda }(t)$ and $I^{T}(t)$ are represented by points constituted by the linear combinations of phasors of its pure components $I_{i}^{\Lambda }(t)(i=1,2,3,4)$ and $I_{i}^{T}(t)(i=1,2,3,4)$. Based on the temporal and spectral profiles of the pure fluorophores in Fig. 6a, the optimal number of clusters to model the data was set to be two and three for temporal and spectral unmixing, respectively. Similar to the experiments conducted on mouse kidney tissue sections and lung cancer pathology slides, we computed the probability estimate of pixels belonging to a particular cluster as $p_{i}^{\Lambda }(i=a,b)$ and $p_{i}^{T}(i=a,b,c)$, such that $\sum_{i}\;p_{i}^{\Lambda }=1$ and $\sum_{i}\;p_{i}^{T}=1$, where a,b,c are indices of classified clusters. Note that the following assumptions are made regarding probability estimates for clusters: $p_{a}^{\Lambda }$ for smad3, $p_{b}^{\Lambda }$ for sma, collagen and $p16,p_{a}^{T}$ for sma, $p_{b}^{T}$ for collagen and smad3, and $p_{c}^{T}$ for p16. By combining the probability estimate with Eq. 18, we can obtain:

(19) $$p_{a}^{\text{Λ}}=f_{3}\;p_{b}^{\text{Λ}}=f_{1}+f_{2}+f_{4}\;p_{a}^{\text{T}}=f_{1}\;p_{b}^{\text{T}}=f_{2}+f_{3}\;p_{c}^{T}=f_{4}.$$

Using this equation, we can calculate the amplitude fractions of the components in the mixture.

### SPAD histograms post-processing

Processing the raw histograms from SPAD involves three steps: background subtraction, delay correction, and nonlinear correction (Fig. S8a). First, in the background subtraction step, we collected the background signal under the same condition as experiments, which results from the dark count of the SPAD camera and stray light from the environment. The resulting background histogram was then subtracted from the raw histogram. Figure S8b shows the histograms after subtraction at two representative pixel locations, indicating that the zero references of histograms of the pixels are not aligned due to the delays in the FPGA from the input to the delay line not being matched [74]. Delay calibration was then performed to correct for the misalignment of histograms. In the delay calibration step, to measure the zero-reference bins of each pixel, we shined a picosecond laser beam onto the pixels and registered the start of the event at each pixel as the zero-reference bin location. Figure S8c presents the histograms at the pixels in Fig. S8b, indicating the zero reference at the maximum bins. The aligned histograms are shown in Fig. S8d after shifting by the zero reference bin values. After the delay correction, a nonlinear correction was conducted to smooth the non-linearities inherent in the delay chains [75]. Using non-time-correlated uniform illumination to the linear SPAD array, we collected a sufficient amount of histograms (∼100) and processed the resultant averaged histogram using histogram equalization to create a uniform histogram [75]. A correction matrix was computed and stored for each pixel during the histogram equalization. Multiplying the correction matrix with raw histograms yields smoothed histograms. Figure S8e shows the raw histogram and smoothed histogram for a 2.5ns period under uniform illumination, and Fig. S8f shows the smoothed histogram after multiplication with the correction matrix.

### Ground truth lung organoid imaging using a confocal fluorescence microscope

To show the ground truth locations of individual biomarkers in our lung organoid experiment, we cultured another four sets of lung organoids under the same condition and labeled them with individual fluorophores. We then imaged the organoids using a standard confocal fluorescence microscope (Zeiss LSM 880 Confocal). Figure S9 presents the color-coded images, which display a similar appearance to our LIFT-sFLIM results.

### Sample preparation

#### Mixed fluorescent beads

To create a mixed fluorescent bead sample, we embedded three types of fluorescent beads (F8858, C16509, F8831, Thermo Fisher) into agarose gels. First, we diluted the bead suspensions and sonicated them. Then, we pipetted 10uL of the 4um bead suspension $(∼5.7\times 10^{7}\;\text{beads}/mL),10\mu L$ of the 6μm bead suspension $\left(∼1.7\times 10^{7}\;\text{beads}/mL\right)$, and 10uL of the 10μm bead suspension $\left(∼3.6\times 10^{6}\;\text{beads}/mL\right)$ into 10mL of PBS (10010023, Thermo Fisher) for each type of bead. We mixed 100μL of the diluted 4μm bead solution, 100μL of the diluted 6μm bead solution, and 100μL of the diluted 10μm bead solution to create the final mixed beads solution, which contained approximately $1.9\times 10^{4}\;4\;\mu m\;\text{beads}/mL,5.6\times 10^{3}\;6\;\mu m\;\text{beads}/mL$, and $1.2\times 10^{3}\;10\;\mu m\;\text{beads}/mL$.

We prepared the agarose gel by making a 1% [weight/volume] solution of low melting point agarose (A6013, Sigma-Aldrich) in PBS, heating it until it completely dissolved, and cooling it down to approximately $40^{\circ }C$. We added 2.5μL of the mixed beads solution to 400μL of the agarose solution. After sonication, we added a 50μL drop of the mixture onto a glass bottom dish (P35G-1.5-14-C, Mattek) and allowed it to solidify for a few minutes. Finally, we imaged the ∼1mm thick gel, which contained immobilized fluorescent beads, using the methods described in the main text.

#### Distal lung organoid preparation

We used a hydrostatic droplet generator to fabricate alginate microbead scaffolds with an average diameter of 100μm, which mimics the size of pulmonary alveoli. After generating the microbeads, we coated them with collagen I (354249, Corning) and dopamine (H8502, Sigma) in a two-step process to functionalize them for cell culture. The detailed protocol for alginate bead generation and functionalization can be found in [57].

Human primary adult normal lung fibroblasts were isolated from distal lung tissue from a de-identified healthy donor (65-year-old, male, Caucasian, non-smoker, non-alcoholic) procured from the International Institute for the Advancement of Medicine (IIAM). Human lung tissue was procured under the UCLA-approved IRB protocol #16-000742. The fibroblast (crawled out population) and epithelial (MACS sorted EpCAM^+^ population) were isolated from the distal tissue and used in this study.

To develop the 3D model, we used a high aspect ratio vessel (HARV) bioreactor vessel (model: RCCS-4H; Synthecon, Houston, Texas) of 2mL volume and added 0.5mL of functionalized microbeads and 1.5mL of media containing a total of 1 million cells (epithelial:fibroblast=1:1). The vessel was screwed into the bioreactor base and rotated for 48h to allow optimum cell-bead adherence. After 48h, the cell-coated bead solution was aliquoted 100μL per well in a glass-bottom 96-well plate (P96-1.5H-N, Cellvis) and the plate was briefly centrifuged (1000g X 2min) to settle the cells/ beads at the bottom of the plate. An additional 150μL media was added to each well. The plate was then kept inside an incubator $\left(37^{\circ }C,5\%{CO}_{2},95\%RH\right)$ and monitored for the formation of self-organized 3D structures. Within the next 72h, the fully-formed 3D co-culture organoids with micro-alveolar structures were observed in each well.

## Data availability

The data of this study is available at https://github.com/iOpticsLab/LIFT-FLIM

## Code availability

The data of this study is available at https://github.com/iOpticsLab/LIFT-FLIM
